# Microbiota composition of simultaneously colonized mice housed under either a gnotobiotic isolator or individually ventilated cage regime

**DOI:** 10.1038/srep42245

**Published:** 2017-02-07

**Authors:** Randi Lundberg, Martin I. Bahl, Tine R. Licht, Martin F. Toft, Axel K. Hansen

**Affiliations:** 1 Department of Veterinary Disease Biology, Faculty of Health and Medical Sciences, University of Copenhagen, 1871 Frederiksberg C, Denmark; 2 Internal Research and Development, Taconic Biosciences, 4623 Lille Skensved, Denmark; 3 National Food Institute, Technical University of Denmark, 2860 Søborg, Denmark

## Abstract

Germ-free rodents colonized with microbiotas of interest are used for host-microbiota investigations and for testing microbiota-targeted therapeutic candidates. Traditionally, isolators are used for housing such gnotobiotic rodents due to optimal protection from the environment, but research groups focused on the microbiome are increasingly combining or substituting isolator housing with individually ventilated cage (IVC) systems. We compared the effect of housing systems on the gut microbiota composition of germ-free mice colonized with a complex microbiota and housed in either multiple IVC cages in an IVC facility or in multiple open-top cages in an isolator during three generations and five months. No increase in bacterial diversity as assessed by 16S rRNA gene sequencing was observed in the IVC cages, despite not applying completely aseptic cage changes. The donor bacterial community was equally represented in both housing systems. Time-dependent clustering between generations was observed in both systems, but was strongest in the IVC cages. Different relative abundance of a Rikenellaceae genus contributed to separate clustering of the isolator and IVC communities. Our data suggest that complex microbiotas are protected in IVC systems, but challenges related to temporal dynamics should be addressed.

Robust systems for testing and validation of hypotheses addressing host-microbiome interactions are crucially important in these still early days within the field, where considerable ambiguity exists regarding methodology, and processing and interpretation of data. Animal models as natural complex systems for microbiome hypothesis-testing have the advantage of enabling the generation of gnotobiotic animals, including germ-free (GF) mice, while exercising control of environmental factors such as housing and husbandry. Due to the risk of introducing random microbes from the environment and caretakers, isolator housing and aseptic husbandry practices are commonly used for microbiome studies in rodents. Isolator housing is considered the gold standard for gnotobiotic animals as these require stringent protection from the surroundings to avoid contamination and compositional shifts of the microbiota. However, there is a need for high throughput methods in which several microbial consortia can be evaluated in *in vivo* test systems within a reasonable time and cost. The use of isolators, which are expensive, difficult to handle and potentially problematic from an experimental design point of view if study groups are contained within separate isolators of their own, is impairing this objective. Previous studies have shown that it is possible to maintain GF mice in individually ventilated cage (IVC) systems for 3–12 weeks^1^,^2^, and that mice carrying unwanted viral and parasitic agents in IVC cages (IVCs) did not cross-contaminate non-infected mice in the same IVC rack^3^. Furthermore, IVC housing has been shown not to increase intercage^4^ or interindividual^5^ variation. However, it is not known if IVC caging increases temporal shifts in the microbiota composition compared to in an isolator. This is relevant to know for breeding facilities, as wells as for experimental facilities, aiming for maintaining mouse cohorts with a stable complex microbiota over time. Accordingly, we colonized GF parent mice with a complex specific pathogen free (SPF) microbiota, housed them in IVCs or in an isolator, and bred them to pass the microbiota on to the offspring. We used clean, but not completely aseptic, cage changing procedures in the IVC system to make the approach simple to work with and broadly applicable. We hypothesized, that the IVCs could be protected from environmental contamination to an extent where this would not cause major compositional shifts in the gut microbiota compared to the gnotobiotic isolator over a period of five months. We based this hypothesis on the assumption that a complex microbiota, once stabilized in the mice, would be relatively resistant to compositional changes caused by the potential introduction of low abundance contaminating bacteria happening during husbandry procedures, such as cage changes.

## Materials and Methods

The experiments were carried out in accordance with the EU directive 2010/63/EU and the Danish Animal Experimentation Act (LBK 474 from 15/05/2014) and were approved by the Danish Animal Experimentation Inspectorate (Ministry of Environment and Food in Denmark) according to license no. 2012-15-2934-00256.

### Microbiota colonization and housing

**Figure 1:**
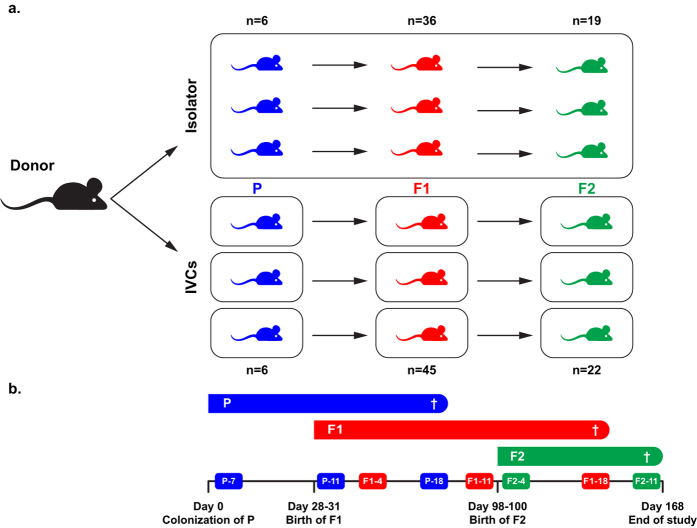
Study design and timeline. (**a**) Study design. Colonic contents from C57BL/6NTac donor mice were used for inoculating germ-free Tac:SW parental (P) mice by oral gavage. P mice were housed in a flexible film isolator or in individually ventilated cages (IVCs). Mice were bred to include P, F1 and F2 generations in the study. The figure is not reflecting the actual numbers of cages. (**b**) Timeline of breeding and fecal samplings. Boxes on the timeline illustrate when fecal samples for 16S rRNA gene sequencing were collected, e.g., P-7 = fecal sampling from P mice 7 weeks old. † = termination of group.

Eight female and four male GF Tac:SW mice (Taconic Biosciences, Germantown, US), hereafter referred to as parental mice (P), arrived at our facility in Denmark when 6 weeks old. Immediately after arrival (Day 0), P mice were colonized by oral gavage with a murine gut microbiota suspension (inoculum). The microbiota donor mice were two female (11 weeks of age) and two male (10 weeks of age) C57BL/6NTac donor mice from a barrier geographically distinct from the IVC barrier used for the study. Donor mice were housed with chlorinated tap water and the same type of bedding, nesting material and gnawing blocks as the recipients housed in the isolator and IVCs, whereas the diet was NIH-31M (Altromin Spezialfutter GmbH & Co. via Brogaarden, Lynge, Denmark), i.e., different from the recipients’ (described below). The inoculum was prepared by homogenizing colonic contents in sterile 25% glycerol (Merck Millipore, Darmstadt, Germany) and frozen at −80 °C until use. The colonization procedure was performed in a biosafety cabinet decontaminated with 1:5:1 Clidox®-S (Indulab, Gams, Switzerland) by a person wearing sterile gown, gloves, face mask and hair cover. P mice were aseptically ear notched for identification, randomized, and divided into two groups and transferred to housing option 1) isolator or 2) IVCs (Fig. 1). After breeding (see below), the study included in total six P mice per group, 36 isolator F1 mice, 45 IVC F1 mice, 19 isolator F2 mice and 22 IVC F2 mice. Initially, P mice were cohoused two-by-two, and breeding trios of two females and one male created after acclimatization. P males were single housed after gestation was confirmed. Three days before births, P females were single-housed. After weaning, P females were cohoused two-by-two again. F1 and F2 pups were socially housed two-five mice/cage. Total number of cages were 10 cages with F1 mice plus six cages with F2 mice in the isolator, and 10 cages with F1 mice plus eight cages with F2 mice in the IVC system. Mice were euthanized as per Fig. 1 by 100% carbon dioxide inhalation by gradual fill of the chamber, and death was confirmed by cervical dislocation.

#### Isolator

The P mice were transferred to a sterile flexible film isolator (CBC, Madison, WI), divided by sex and housed in Eurostandard Type II L polycarbonate cages (Tecniplast, Varese, Italy). The isolator was tested sterile for three consecutive weeks by aerobic and anaerobic culturing of bacteria and fungi before animals and materials were introduced. Drinking water was autoclaved bottled non-chlorinated tap water *ad libitum*. The isolator had approximately eight air changes/hour. Autoclaved supplies were sterilely introduced to the isolator when necessary, but never more than once a week.

#### IVCs

The P mice were divided by sex, transferred to autoclaved assembled polysulfone IVC cages (NexGen Mouse Easy IVC, Allentown Inc., Allentown, NJ) with bedding and nesting material and placed in an IVC rack in an IVC barrier unit with Taconic’s Restricted Flora^TM^ Health Standard. Drinking water *ad libitum* was bottled and came from the same tap water source as for the isolator, but was chlorinated to reach a concentration of 5–10 ppm to avoid bacterial growth in the piping system installed in the IVC barrier unit. Cages had 50 air changes/hour. Cage changes were done by one person wearing autoclaved, but not sterilely packed, personal protective equipment (gown, two layers of gloves, mask and hair cover) in a biosafety cabinet decontaminated with 1:5:1 Clidox®-S (Indulab) in the following way: The cage was removed from the rack and placed in the biosafety cabinet and the lid was removed without touching the insides of the cage. The gloves were sprayed with 70% ethanol and the mice were moved to the clean cage with forceps sterilized with Clidox 1:5:1. Fecal samples from individual mice were obtained by manual restraint of the scruff of the neck. Thorough disinfection of gloves between handling of each mouse was done with 70% ethanol, which was allowed to evaporate before handling the next animal. Autoclaved food and gnawing blocks were added to the cage which was then returned to the rack. No additional decontamination of the biosafety cabinet was done between cages.

#### Common conditions for isolator and IVCs

All cages were changed weekly and equipment, food and materials were sterilized by autoclaving prior to cage changes. Bedding was JELUXYL HW 300/500 (JELU WERK, Rosenberg, Germany), nesting material Soft Paper Wool (Brogaarden) and gnawing blocks Aspen size S (Tapvei, Harjumaa, Estonia). Light/dark cycle was 12/12 hours, the temperature 20–23 °C, and the ambient relative humidity was in the range of 45–65%. The diet was *ad libitum* ssniff® M-Z Low-Phytoestrogen V1154-3 breeding diet for mice (ssniff Spezialdiäten, GmbH, Kiel, Germany).

### Breeding

Four days after arrival (Day 4), a handful of dirty bedding was transferred from P males to P females to facilitate synchronization of the oestrus cycle. On Day 7, breeding trios of two females and one male were set up. All P females gave birth between Day 28–31. F1 litter sizes were 10–12 pups in the isolator and 11–15 in the IVCs. Eight and five F1 mice from each housing system, respectively, were found dead before weaning or euthanized for animal welfare reasons. The F1 pups were weaned and ID marked by ear notching when 21–24 days old (Day 52). Each sex from same litter was subdivided to at least two cages when possible to enable control of the experimental factors “litter”, “sex” and “cage”. On Day 77, when the F1 mice were 7 weeks old, two breeding duos for each housing system were set up using the same procedure with prior moving of dirty bedding from males to females as described for P mice. The F2 pups were born on Day 98–100, litter sizes were 9–10 pups in the isolator and 10–13 in the IVCs. One F2 pup from the IVCs was euthanized before weaning for animal welfare reasons.

### Fecal sampling

Individual fecal samples were obtained by restraint and letting the mouse defecate directly into autoclaved 1.5 ml Safe-Lock Eppendorf® microcentrifuge tubes (Buch & Holm A/S, Herlev, Denmark) from P mice aged 7 weeks (one week post-colonization), 11 and 18 weeks; from F1 mice aged 4, 11 and 18 weeks; and from F2 mice aged 4 and 11 weeks (Fig. 1). Fecal samples were stored at −20 °C within 30 minutes after sampling, and later moved to long term storage at −80 °C. Fecal donor material stored at −80 °C without glycerol was used for the subsequent sequencing.

### DNA isolation

All samples were randomized prior to DNA isolation. DNA from 1–3 fecal pellets/sample was isolated using PowerLyzer® PowerSoil® DNA Isolation Kit (MO BIO Laboratories, Inc., Carlsbad, CA, USA) according to the manufacturer’s instructions with the following modifications: Bead beating was done at 30 cycles/s for 2 × 5 minutes with 10 minutes rest in-between on a Mixer Mill MM 300 (Retsch GmbH, Haan, Germany), and centrifuging was done for 1 minute at 13000 rpm. The DNA concentration was quantified using Qubit® 2.0 Flurometer and dsDNA HS assay kit (ThermoFischer Scientific, Naerum, Denmark). DNA template for PCR was prepared by diluting the DNA to 5 ng/μl. Samples with concentrations <5 ng/μl were left undiluted.

### Library building

The V3 region of the 16S rRNA gene was amplified by PCR as previously described^6^. The amplicons were purified using HighPrep^TM^ PCR Clean Up System (MagBio Genomics Inc., Rockville, MD, USA) according to the manufacturer’s protocol. The DNA libraries were multiplexed in batches of 89 in an equimolar ratio obtained by measuring the DNA concentration using Qubit® as described for DNA isolation, and then mixing the same amount of DNA to each library. The multiplexed libraries were stored at −20 °C until sequencing.

### 16S rRNA gene sequencing and data processing

The 16S rRNA gene libraries were sequenced on the Ion PGM^TM^ System using a 318-chip, the Ion PGM^TM^Template OT2 200 Kit and the Ion PGM^TM^ Hi-Q^TM^Sequencing Kit (ThermoFischer Scientific). Data were imported to CLC BIO Genomic Workbench vs. 7.0 (CLC bio, Qiagen, Aarhus, Denmark) where reads were de-multiplexed and trimmed to remove primers, barcodes, low quality sequences (quality score = 0.05), ambiguous nucleotides (maximally 2 allowed) and reads below 110 bp and above 180 bp. Further data processing was performed according to the pipeline for Ion Torrent data from the Brazilian Microbiome Project^7^ with minor modifications. Briefly, Operational Taxonomic Units (OTUs) were picked de novo using UPARSE^8^ with a maximum expected error (maxee) rate of 3.5 and no truncation of reads. Chimera filtering was done using the rdp_gold.fa database as reference^9^. Taxonomy was assigned in the software package Quantitative Insights Into Microbial Ecology^10^ (QIIME) version 1.9.1 to OTUs with 97% similarity using the Silva 111 reference database^11^. Samples with less than 1300 reads were filtered from the total set of 227 samples. The resulting set of 222 samples (Isolator: 88; IVCs: 132; Inoculum: 2 PCR duplicates) had a mean of 27632 reads/sample (Min: 1318, Max: 88078; SD: 15069) and 2534 OTUs after discarding singleton OTUs (i.e., observed only once). Normalization for calculation of alpha diversity, colonization efficiency and differential abundance analysis was done by rarefying the OTU table to 1200 reads/sample, corresponding to ~90% of the sample with fewest reads. The script alpha_diversity.py was used to compute observed species (richness) and Shannon’s diversity index. The workflow beta_diversity_through_plots.py was used to make weighted and unweighted UniFrac distance matrices with a depth coverage of 1200 reads/sample^12^, perform Principal Coordinates Analysis (PCoA) and generate 3D PCoA plots in EMPeror^13^. For description of relative abundance, a cut-off threshold at 1% was set, meaning that taxa with abundance <1% was collapsed. We interpreted the database output genus Candidatus *Arthromitus*, present in the inoculum and both housing systems, as Segmented Filamentous Bacteria (SFB). Candidatus *Arthromitus* is probably classified incorrectly in the database and should rightfully be called Candidatus *Savagella* or SFB, as the arthropod bacterium Candidatus *Arthromitus* truly belongs to Lachnospiraceae^14^, while the vertebrate SFB belongs to the family of Clostridiaceae^15^. Thus, as the Candidatus *Arthromitus* of our study was classified within Clostridiaceae it should be interpreted as Candidatus *Savagella*, i.e., the vertebrate SFB.

### Statistical methods

Model assumptions for alpha diversity data were assessed by residual plots and Anderson-Darling normality test, and data were analysed by Student’s t-test or one-way ANOVA with Tukey’s pairwise comparisons as appropriate in Minitab®17 Statistical Software (Minitab Ltd., Coventry, UK). GraphPad Prism 6 (GraphPad Software, La Jolla, CA, USA) was used to create alpha diversity plots. For calculating colonization efficiency, a cut-off of 0.1% abundance was applied. No filtering out of taxa that were present in only a few samples was employed for the analysis of potential contamination, as this was deemed most appropriate in terms of testing our hypothesis. Differential abundance analysis was done on tables with OTUs summarized on genus level and filtered from OTUs present in less than 25% of the samples by Kruskal-Wallis test with 1000 permutations and Bonferroni correction for multiple comparisons using QIIME’s script group_significance.py. The script compare_categories.py was used to determine beta diversity clustering statistically on filtered distance matrices allowing for pair-wise comparisons by the method ANOSIM (analysis of similarities) using 999 permutations. We considered an R-value of 0.75–1 as strong separation, 0.25–0.75 as moderate separation, and <0.25 as low separation in the ANOSIM tests. Testing UniFrac distance differences to the inoculum was done by the script make_distance_comparison_plots.py using the built in Student’s t-test with Bonferroni correction. All analyses were performed on a 95% significance level. A p-value > 0.05 and <0.08 was considered a tendency.

## Results

### Colonization was equally efficient in isolators and IVCs, but unique taxa were detected in both systems compared to the inoculum

**Figure 2:**
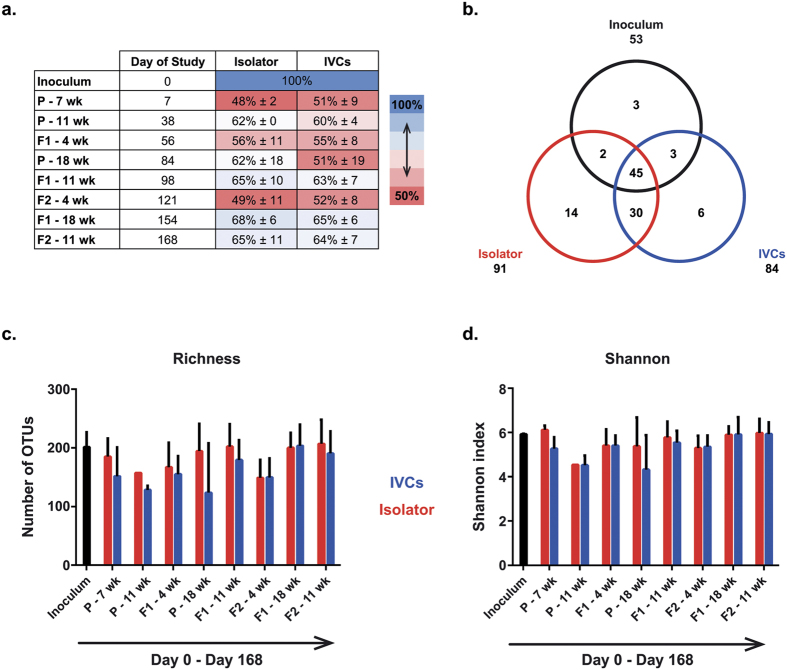
Colonization efficiency and alpha diversity of the microbiota of mice from different generations. (**a**) Heat map of colonization efficiency of each generation compared to inoculum. Detected taxa on genus level in the inoculum correspond to 100%, and the mean percentage with s.d. in each housing system is shown for each generation and age time point. There were no differences between the housing systems, but colonization efficiency for P at 7 weeks and F1 and F2 at 4 weeks were significantly lower than the other time points in both housing systems (Supplementary Table S1). (**b**) Venn diagram showing number of unique genus level taxa shared between the housing systems and the inoculum. (**c+d**) Richness (number of OTUs not summarized to a specific taxonomic level) (**c**) and Shannon index (**d**) of inoculum and fecal samples from each generation and age sorted per the timeline of the study (Day 0-Day 168). There were no differences between the housing systems at any time point (t-test; bars are s.d.). P = parent mice inoculated at 6 weeks of age; F1 and F2 = offspring generations born with the microbiota.

Colonization efficiency was calculated in each generation at selected ages by identifying number of taxa on genus level in the recipient animals and determining the fraction compared to the taxa in the inoculum. No significant differences were found in colonization efficiency between the two housing systems at any time point (Fig. 2a). The colonization efficiency of the P mice determined seven days after inoculation was 48% ± 2 and 51% ± 9 in the isolator and IVCs, respectively. The colonization efficiency in F1 offspring at 18 weeks of age was higher than in the P mice seven days after inoculation in the isolator (68% ± 6; p = 0.038), and tended to be higher in the IVCs (65% ± 6; p = 0.061). We also observed that the colonization efficiency of samples from pups aged 4 weeks were significantly lower than in samples from older animals (Supplementary Table S1). Next, we counted the number of overlapping taxa with the inoculum of all samples to assess whether more taxa would be detected in the less protected IVCs over the study period of five months. A total of 53 genus level taxa (mean relative abundance 1.9% ± 7.1) were identified in the inoculum. Of these, 47 were recovered in the isolator and 48 in the IVCs, with 45 being common between the two. Additional 50 sparsely dispersed and very low abundance (relative abundance 0.004% ± 0.03, corresponding to 2–20 reads/sample; not shown) taxa were found in the isolator and IVCs, which were not detected in the inoculum (Fig. 2b).

### Richness and Shannon indices were not affected by housing type but were age dependent

**Figure 3:**
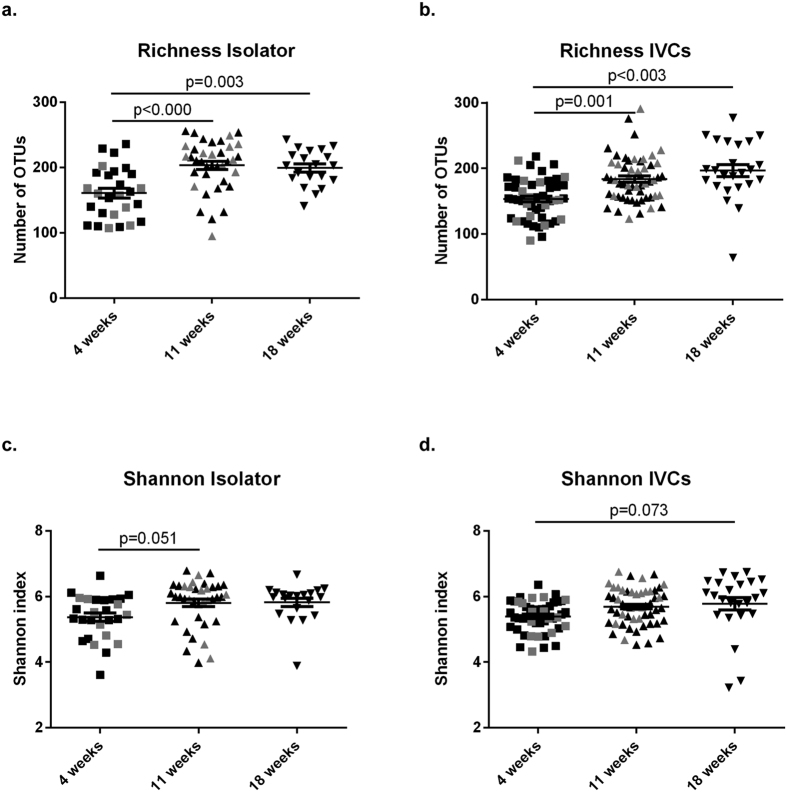
Alpha diversity of the gut microbiota at different ages. Richness (**a+b**) and Shannon index (**c+d**) of fecal samples from F1 and F2 mice aged 4, 11, and 18 weeks. The 4 and 11 week age time points represent cumulated samples from F1 (black symbols) and F2 (grey symbols), as these were not statistically different. 18 weeks represent F1 only, as the study did not include F2 mice aged 18 weeks (ANOVA with Tukey’s pairwise comparisons; bars are s.e.m.).

To further address the hypothesis that IVC housing would not significantly increase the number of taxa, we investigated richness and Shannon’s diversity index by housing type and age. There were no statistically significant differences of the two alpha diversity-metrics between the housing systems at any age (Fig. 2c+d). As the gut microbiota is known to go through an establishment phase with increasing diversity after weaning^16^, we investigated the age effect in each housing system separately (Fig. 3; Supplementary Table S2). Indeed, we observed lower richness and a small trend for lower Shannon diversity in samples from mice when they were weaned at 4 weeks of age compared to mice sampled at 11 and 18 weeks of age in both housing systems irrespective of generation. There were no differences in richness or Shannon index according to sex (Supplementary Figure S1).

### Temporal composition dynamics were strongest in the IVCs

**Figure 4:**
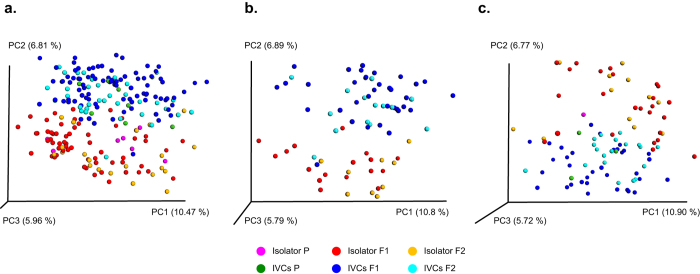
Unweighted UniFrac PCoA plots showing gut microbiota beta diversity of isolator- and IVC-housed mice over three generations. (**a**) Fecal samples from all isolator and IVC samples (i.e., P aged 7, 11 and 18 weeks, F1 aged 4, 11 and 18 weeks, and F2 aged 4 and 11 weeks) coloured per generation (**b**) Fecal samples from F1 mice aged 4 weeks only. F1 and F2 displayed low separation in both housing systems (Unweighted PCoA; Isolator: p = 0.008; R = 0.21. IVCs: p = 0.002; R = 0.23) (**c**) Fecal samples from F1 mice aged 11 weeks only. There was no significant separation of the F1 and F2 generation at 11 weeks of age in either housing system. P = parent mice inoculated at 6 weeks of age; F1 and F2 = offspring generations born with the microbiota.

To assess changes over time and vertical transmission of the microbiota from parents to the offspring, we explored clustering between the generations at the various ages of each housing system. Distinct clustering within each housing system between generations was not visible in the PCoA plot with samples from all ages (Fig. 4a). However, F1 and F2 displayed a low statistically significant separation in both housing systems at 4 weeks of age (Unweighted, Isolator: p = 0.008; R = 0.21. IVCs: p = 0.002; R = 0.23), but not at 11 weeks of age (Fig. 4b+c; Table 1). Accordingly, we tested differentially abundant taxa and found that there was no significant different distribution of taxa between F1 and F2 in the isolator at either 4 or 11 weeks of age, whereas five and three taxa were different between F1 and F2 in the IVCs at 4 and 11 weeks of age, respectively (Table 2). Due to few samples in the P generation we could not statistically test the P generation at various ages against the offspring generations. Instead, we cumulated all P samples and tested against all F1 or F2 samples irrespective of age and found P to be clearly segregated from F1 and F2 in the IVCs, while only a tendency was reached in the isolator for P compared to F1 and F2 (Table 1).

### Isolator and IVC samples clustered separately from each other and was equally distant from the inoculum

**Figure 5:**
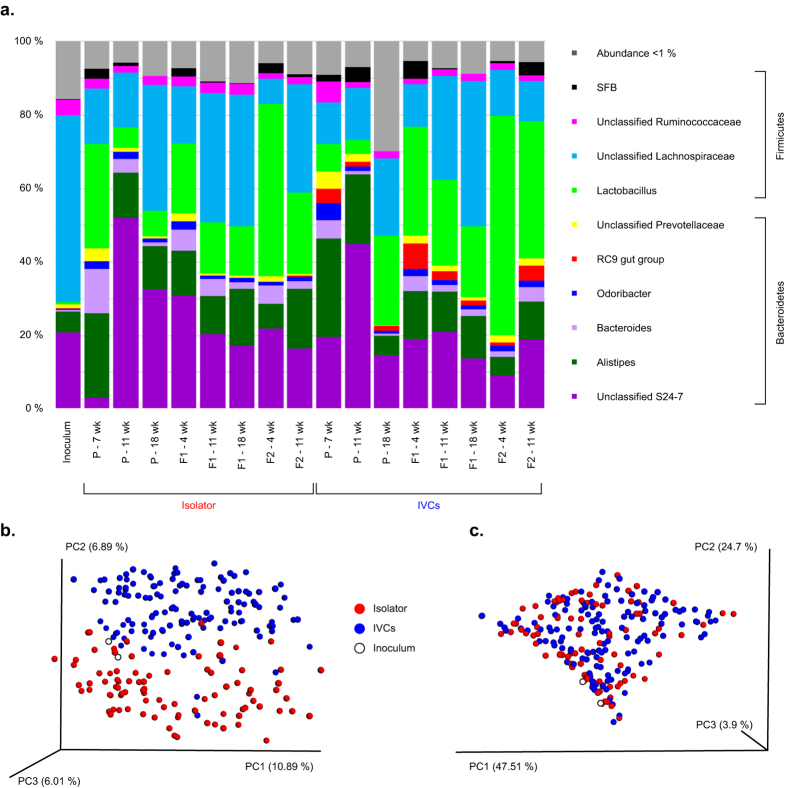
Gut microbiota composition and beta diversity of inoculum, isolator- and IVC-housed mice. (**a**) Relative abundance (%) of the 10 most abundant genus level taxa in inoculum and fecal samples from the isolator and IVCs per generation and age. Taxa with an abundance <1% were collapsed. (**b+c**) Unweighted (**b**) and weighted (**c**) UniFrac PCoA plots of all isolator and IVC samples (i.e., P aged 7, 11 and 18 weeks, F1 aged 4, 11 and 18 weeks, and F2 aged 4 and 11 weeks), and the inoculum in PCR duplicates. Isolator and IVC samples displayed a low to moderate separate clustering from each other (Unweighted: p = 0.001, R = 0.34; Weighted: p = 0.003, R = 0.04). P = parent mice inoculated at 6 weeks of age; F1 and F2 = offspring generations born with the microbiota.

Compared to the inoculum, there was an increase in the relative abundance of the genus *Lactobacillus* in all recipient groups of mice. The unclassified genus “*RC9 gut group*” within the family Rikenellaceae had a low abundance in the inoculum (0.1%) and was only detected in the isolator in the F2 generation. In the IVCs, “*RC9 gut group*” increased its relative abundance remarkably in all generations (Fig. 5a; Supplementary Table S3). The isolator and IVC samples cumulated across age and generations clustered moderately distinct from each other in the qualitative (unweighted) UniFrac PCoA (p = 0.001, R = 0.34; Fig. 5b), and less distinct in the quantitative (weighted) PCoA (p = 0.003, R = 0.04; Fig. 5c). We observed the abundance of eight taxa to differ significantly between the housing systems (Table 3), and all of them were also found in the inoculum. The “*RC9 gut group*” appeared to be one of the main drivers of the clustering between the two housing groups. Compared to the inoculum, the mean UniFrac distance of all IVC samples was no longer than that of the isolator samples (p = 0.14). There were no differences in beta diversity according to sex in either housing system (ANOSIM and differential abundance analysis; Supplementary Figure S1).

## Discussion

By employing clean but not rigorously sterile procedures when changing the IVCs, we have shown that a complex murine microbiota seems to be protected from environmental contamination, as the alpha diversity, although lowest in samples from mice aged 4 weeks, was stable over generations in both housing systems and was not higher in the IVCs. Colonization was equally efficient in both housing systems. However, some low abundance taxa were detected in the recipients, that were not detected in the inoculum. Other fecal transplantation studies in mice using 16S rRNA gene sequencing have similarly reported presence of low abundance taxa in the recipients, which were not detected in the donor communities^17^,^18^,^19^. In our study, we speculate that these taxa were either selectively proliferating in the recipient mice and therefore constituted a larger proportion of the total microbiota in the recipients than in the inoculum, where the levels may have been below the detection limit, or the likelihood of detecting these taxa were higher in the high number of biological replicate samples of the recipient mice compared to the single inoculum. An alternative explanation is that these taxa represented contaminating bacteria that had entered the housing systems. Contamination could potentially have happened during the inoculation process, during cage changes in the IVCs and transport of materials into the isolator, or during handling of samples in the context of sampling, storage, DNA isolation, or PCR library preparation. In either case, the number of potentially contaminating taxa were comparable between the isolator and IVC samples, suggesting that if contamination was a primary cause, it was not related to differences in housing systems, but rather to conditions generic for both systems, e.g. contamination during preparation for PCR. The colonization efficiency (i.e. the fraction of taxa from the inoculum which was recovered in the recipient animals) was lower in the P generation compared to the F1 generation when mice were 18 weeks old. Thus, maybe colonization at 6 weeks of age is not optimal. Previously, it was shown that 3 weeks of age is an efficient time point for fecal transplantation aiming to establish a community similar to the inoculum^20^. Future studies should address if there are benefits of using offspring of inoculated animals rather than the inoculated animals themselves, which would also facilitate the most natural immune shaping, when pups are exposed to the microbes directly after birth^21^. Consistent with the lower alpha diversity of samples from 4 weeks old mice, the colonization efficiency was lower in this age group and of their microbiotas clustered separately compared to the other age time points. The mouse gut microbiota develops from low diversity in suckling mice to the diversity level of the dams around the time of weaning^16^. Additionally, the microbiota of 4 weeks old mice was reported to cluster differently from the same mice aged 12 and 24 weeks^22^. Altogether, this underlines that the murine microbiota around the time of weaning cannot be considered stable.

We noted time-dependent segregation of the microbiota in both housing systems concerning samples from 4 weeks old mice of the F1 and F2 generations. This clustering may be ascribed to the instability of the microbiota at this age. There was also a different clustering between the P generation and the subsequent generations, which was significant in the IVCs and displayed a trend in the isolator. Additionally, we observed that the relative abundance of some genera was different between the F1 and F2 generations both at 4 weeks and 11 weeks of age in the IVCs, implying a generation-wise, or time-dependent, clustering across cages was specifically present in the IVC system. We monitored our mouse cohort for five months, but going forward it would be useful to explore time-wise segregation of complex microbiotas in IVC systems over longer time. It might be that practices such as sharing of dirty bedding between cages or regular re-colonization with the starter microbiota is necessary to minimize compositional shifts in the microbiota over time.

The relative abundance of *Lactobacilli* was, compared to the inoculum, higher in both housing systems. The Lactobacillaceae family, including *Lactobacilli*, are well-known inhabitants of the rodent gut^23^, and *Lactobacilli* have previously been observed to bloom after microbiota transplantation in mice^24^. Why *Lactobacilli* in particular increased its abundance in the recipients is unknown, but maybe transferring bacteria between hosts via the orogastric route provides an opportunistic advantage for specific acidophilic taxa such as *Lactobacilli* in the establishing phase, which is reflected in the offspring generations as well. Differences in diets or other husbandry factors between the donor and recipient mice may also explain why some taxa profoundly increase or decrease in abundance compared to the donor community. Also, the difference between the inbred donor strain and outbred recipient stock could in theory select for differences in the gut microbiota, though environment more than genetics is demonstrated to drive the composition of the microbiota^25^,^26^.

The microbiota of the two housing systems clustered qualitatively different from each other, but were equally close to the donor community. Specifically, the genus “*RC9 gut group*” of the Rikenellaceae family appeared to be a main driver of this clustering. There are several possible explanations for this, which are likely to be intertwined. First, the drinking water in the IVC system was chlorinated. Chlorine is likely to affect the gut microbiota, and may thus have contributed to the different clustering of samples originating from different housing systems. Changing the pH by water treatment regimens such as hydrochloric acid has previously been shown to affect the gut microbiota^27^,^28^, and chlorination specifically was recently reported to alter colonic tumour formation in adenomatous polyposis coli-deficient mice, putatively via changes in the gut microbiota^29^. Second, random community dynamic events happening in the establishment phase may lead to different steady-state communities within the hosts independent of housing system. Third, distinct stress levels in the two housing systems may contribute to clustering between the microbial communities. *Alistipes* (Rikenellaceae), an organism closely related to “*RC9 gut group*” and previously linked to stress^30^, did not differ in abundance in the two housing systems, while Rikenellaceae on the family level has previously been reported to increase its abundance in mice subjected to acute restraint stress^31^. We did not observe other stress indicators, such as different breeding performances or body weights (Supplementary Figure S2), however, primary stress markers such as corticosterone were not assessed. IVC systems have been reported to induce cold stress in mice, but this could be counteracted by providing shelters and sufficient nesting material to facilitate mouse-controlled thermoregulation^32^. The mice in our study had nesting material, but there may be other environmental parameters in the IVC barrier influencing the microbiota via stress-induced pathways, such as noise, different staff, and different ventilation systems. Fourth, the microbiota of all the cages in the isolator could theoretically be subjected to a so-called isolator effect, corresponding to cage effect, i.e., the phenomenon that the microbiota of co-housed laboratory rodents can be more similar to each other than to the microbiota of conspecifics housed in other cages. Cage effect is observed in many studies and is explained by microbe-sharing due to coprophagia and because the confined cage environment permits differential microbiota drifting over time within each cage. Oral gavage with a given specific microbial community was e.g. previously shown insufficient to eliminate cage effect suggesting that stochastic changes over time drives appearance of cage effect^33^. In the isolator, microbe-sharing between cages can happen when gloves and materials stored in the isolator act as fomites, via the air flow and when dust and bedding is whirled around due to activity of the animals. As such, the isolator should be regarded as a single microbiological study unit. In contrast, if intercage decontamination is implemented, each cage in the IVC rack can be viewed as a separate isolator and should accordingly be considered as one microbiological study unit, which are replicated according to the number of replicate cages included in each study group. Hence, seen from a study design point of view there are arguments in favour of IVC housing, if the alternative is that each study group with a designated microbiota is housed in a single isolator due to equipment and space limitations. A follow-up study comparing several isolators populated with mice colonized with the same starter microbiota is necessary to explore the proposed isolator effect.

With the increasing evidence revealing the impact of the barrier-protected microbiota on various mouse and rat disease models^34^ the importance of controlling the laboratory rodent microbiota has become apparent. Various ways of controlling the barrier-protected SPF microbiota of rodent models have been proposed, including standardization of the baseline microbiota, tailoring the microbiota for use in specific models, and characterizing the microbiota and incorporating this information in the statistical data evaluation^35^,^36^. The first approach, standardization of the baseline microbiota, requires a controlled effort starting from the birth of the animals, i.e., typically in the vendor’s facility. Disparity in the microbiota between animal vendors^4^,^37^,^38^, or between rooms in the same experimental SPF facility^4^,^39^, is well described and has been shown to affect the phenotype in several mouse and rat disease models^39^,^40^,^41^,^42^. In fact, the vendor factor has recently been shown to drive gut microbiota composition more than sex, mouse strain, and diet^26^. Even mice received from the same vendor vary between and within batches^43^ and may affect disease expression in a range of animal models^44^,^45^. Hence, there seems to be a rationale at the individual vendor’s level to minimize variation within and between colonies of the same strain or stock. Gnotobiotic isolator housing for large-scale breeding of production colonies is impractical. Alternatively, IVC housing may be applicable for maintaining barrier-protected rodent colonies with standardized, complex microbiotas without causing shifts in the intended composition due to contamination, but the effect over longer time than assessed here should be investigated.

In summary, our findings suggest the use of IVC systems for maintaining rodents with semi-defined complex microbiotas for up to at least five months, which may also imply that a statistically stronger study design can more easily be achieved in microbiome studies compared to using a high number of isolators. However, the time-dependent effect observed in the IVCs could in theory drive disease parameter variation in certain models, and future work should therefore address this. When using IVC systems for projects where a completely gnotobiotic status is imperative, much more complicated sterile procedures than reported here are necessary, e.g., as recently described^2^,^46^. Thus, the purpose and expected susceptibility to microbiota fluctuations should be carefully evaluated before deciding on the best housing system for the purpose.

## Additional Information

**How to cite this article:** Lundberg, R. *et al*. Microbiota composition of simultaneously colonized mice housed under either a gnotobiotic isolator or individually ventilated cage regime. *Sci. Rep.* **7**, 42245; doi: 10.1038/srep42245 (2017).

**Publisher's note:** Springer Nature remains neutral with regard to jurisdictional claims in published maps and institutional affiliations.

## Supplementary Material

Supplementary Information

## Figures and Tables

**Table 1:** Clustering of the gut microbiota of fecal samples from isolator- and IVC-housed mice.

Unifrac distance matrix used for ANOSIM	Group	Hypothesis	p-value	R-value	Degree of separation
**Unweighted**	**Isolator**	P vs. F1 (all ages)	0.065(*)	0.20	low
		P vs. F2 (all ages)	0.077(*)	0.15	low
		F1 vs. F2 (4 weeks)	0.008*	0.21	low
		F1 vs F2 (11 weeks)	0.636	0.03	none
**Weighted**		P vs. F1 (all ages)	0.135	0.11	none
		P vs. F2 (all ages)	0.335	0.02	none
		F1 vs. F2 (4 weeks)	0.002*	0.29	moderate
		F1 vs. F2 (11 weeks)	0.491	0.00	none
**Unweighted**	**IVCs**	P vs. F1 (all ages)	0.002*	0.40	moderate
		P vs. F2 (all ages)	0.015*	0.28	moderate
		F1 vs. F2 (4 weeks)	0.002*	0.23	low
		F1 vs F2 (11 weeks)	0.832	0.05	none
**Weighted**		P vs. F1 (all ages)	0.001*	0.37	moderate
		P vs. F2 (all ages)	0.001*	0.37	moderate
		F1 vs. F2 (4 weeks)	0.001*	0.50	moderate
		F1 vs. F2 (11 weeks)	0.690	0.03	none

Statistical tests between generation and age time points within the two housing systems based on weighted and unweighted UniFrac distance matrices. We considered a p-value > 0.05 and <0.08 as a tendency and is designated with (*). P-values < 0.05 are designated with * (ANOSIM with 999 permutations).

**Table 2:** Differentially abundant taxa between the generations of IVC-housed mice.

Phylum	Class	Order	Family	Genus	p-value	F1 abund.	F2 abund
**IVCs 4 weeks**							
Bacteroidetes	Bacteroidia	Bacteroidales	Rikenellaceae	Alistipes	<0.01	13.04%	4.99%
				RC9 gut group	<0.001	6.88%	0.87%
Firmicutes	Bacilli	Bacillales	Staphylococcaceae	Staphylococcus	<0.01	0.00%	0.37%
		Lactobacillales	Lactobacillaceae	Lactobacillus	<0.001	29.95%	59.94%
	Clostridia	Clostridiales	Clostridiaceae	SFB	<0.001	4.78%	0.56%
**IVCs 11 weeks**							
Bacteroidetes	Bacteroidia	Bacteroidales	Rikenellaceae	Alistipes	0.019	11.07%	17.23%
Firmicutes	Clostridia	Clostridiales	Clostridiaceae	SFB	<0.001	0.25%	1.21%
Proteobacteria	Deltaproteobac.	Desulfovibrionales	Desulfovibrionaceae	Desulfovibrio	0.015	0.45%	1.00%

Taxa of fecal samples from IVC mice aged 4 and 11 weeks had different abundances in F1 and F2 generations. Abundance is given as mean abundance of the group in percent (Kruskal-Wallis with 1000 permutations and Bonferroni correction test). Abund. = Abundance.

**Table 3:** Differentially abundant taxa in isolator vs. IVC samples.

Phylum	Class	Order	Family	Genus	p-value	Isolator abund.	IVCs abund.
Bacteroidetes	Bacteroidia	Bacteroidales	Rikenellaceae	RC9 gut group	<0.001	0.06%	3.18%
Firmicutes	Clostridia	Clostridiales	Unclassified	Unclassified	0.002	1.13%	0.56%
			Lachnospiraceae	Roseburia	0.001	0.12%	0.05%
			Ruminococcaceae	Unclassified	0.009	2.51%	1.86%
				Flavonifractor	0.002	0.11%	0.05%
				Unclassified	<0.001	1.01%	0.43%
				Ruminococcus	<0.001	0.19%	0.02%
Tenericutes	Mollicutes	RF9	Unclassified	Unclassified	0.016	0.23%	0.13%

Fecal samples from isolator and IVC-housed mice had different abundances of eight taxa. Abundance is the mean abundance of the group in percent (Kruskal-Wallis with 1000 permutations and Bonferroni correction test). Abund. = Abundance.

## References

[b1] Hecht G. . A simple cage-autonomous method for the maintenance of the barrier status of germ-free mice during experimentation. Lab. Anim. 48, 292–7 (2014). 25097255 10.1177/0023677214544728

[b2] Paik J. . Potential for using a hermetically-sealed, positive-pressured isocage system for studies involving germ-free mice outside a flexible-film isolator. Gut Microbes 6, 255–65 (2015). 26177210 10.1080/19490976.2015.1064576 PMC4615381

[b3] Brielmeier M. . Microbiological monitoring of laboratory mice and biocontainment in individually ventilated cages: a field study. Lab. Anim. 40, 247–60 (2006). 16803642 10.1258/002367706777611497

[b4] Hufeldt M. R., Nielsen D. S., Vogensen F. K., Midtvedt T. & Hansen A. K. Variation in the gut microbiota of laboratory mice is related to both genetic and environmental factors. Comp. Med. 60, 336–47 (2010). 21262117 PMC2958200

[b5] Thoene-Reineke C. . Composition of intestinal microbiota in immune-deficient mice kept in three different housing conditions. PLoS One 9, e113406 (2014). 25401702 10.1371/journal.pone.0113406 PMC4234647

[b6] Christensen E. G., Licht T. R., Leser T. D. & Bahl M. I. Dietary xylo-oligosaccharide stimulates intestinal bifidobacteria and lactobacilli but has limited effect on intestinal integrity in rats. BMC Res. Notes 7, 660 (2014). 25238818 10.1186/1756-0500-7-660 PMC4179812

[b7] Pylro V. S. . Data analysis for 16S microbial profiling from different benchtop sequencing platforms. J. Microbiol. Methods 107, 30–7 (2014). 25193439 10.1016/j.mimet.2014.08.018

[b8] Edgar R. C. UPARSE: highly accurate OTU sequences from microbial amplicon reads. Nat. Methods 10, 996–8 (2013). 23955772 10.1038/nmeth.2604

[b9] Edgar R. C., Haas B. J., Clemente J. C., Quince C. & Knight R. UCHIME improves sensitivity and speed of chimera detection. Bioinformatics 27, 2194–200 (2011). 21700674 10.1093/bioinformatics/btr381 PMC3150044

[b10] Caporaso J. G. . QIIME allows analysis of high-throughput community sequencing data. Nat. Methods 7, 335–6 (2010). 20383131 10.1038/nmeth.f.303 PMC3156573

[b11] Quast C. . The SILVA ribosomal RNA gene database project: improved data processing and web-based tools. Nucleic Acids Res. 41, D590–6 (2013). 23193283 10.1093/nar/gks1219 PMC3531112

[b12] Lozupone C. & Knight R. UniFrac: a new phylogenetic method for comparing microbial communities. Appl. Environ. Microbiol. 71, 8228–35 (2005). 16332807 10.1128/AEM.71.12.8228-8235.2005 PMC1317376

[b13] Vázquez-Baeza Y., Pirrung M., Gonzalez A. & Knight R. EMPeror: a tool for visualizing high-throughput microbial community data. Gigascience 2, 16 (2013). 24280061 10.1186/2047-217X-2-16 PMC4076506

[b14] Thompson C. L., Vier R., Mikaelyan A., Wienemann T. & Brune A. ‘Candidatus Arthromitus’ revised: segmented filamentous bacteria in arthropod guts are members of Lachnospiraceae. Environ. Microbiol. 14, 1454–65 (2012). 22436008 10.1111/j.1462-2920.2012.02731.x

[b15] Thompson C. L., Mikaelyan A. & Brune A. Immune-modulating gut symbionts are not ‘Candidatus Arthromitus’. Mucosal Immunol. 6, 200–201 (2013). 23013646 10.1038/mi.2012.91

[b16] Pantoja-Feliciano I. G. . Biphasic assembly of the murine intestinal microbiota during early development. ISME J. 7, 1112–1115 (2013). 23535917 10.1038/ismej.2013.15 PMC3660675

[b17] Wos-Oxley M. . Comparative evaluation of establishing a human gut microbial community within rodent models. Gut Microbes 3, 234–49 (2012). 22572831 10.4161/gmic.19934 PMC3427216

[b18] Turnbaugh P. J. . The effect of diet on the human gut microbiome: a metagenomic analysis in humanized gnotobiotic mice. Sci. Transl. Med. 1, 6ra14 (2009). 10.1126/scitranslmed.3000322 PMC2894525 20368178

[b19] Zhang L. . Environmental spread of microbes impacts the development of metabolic phenotypes in mice transplanted with microbial communities from humans. ISME J., doi: 10.1038/ismej.2016.151 (2016). PMC5322303 27858930

[b20] Hansen C. H. F. . Patterns of early gut colonization shape future immune responses of the host. PLoS One 7, e34043 (2012). 22479515 10.1371/journal.pone.0034043 PMC3313961

[b21] Lundberg R., Toft M. F., August B., Hansen A. K. & Hansen C. H. F. Antibiotic-treated versus germ-free rodents for microbiota transplantation studies. Gut Microbes 7, 68–74 (2016). 26744774 10.1080/19490976.2015.1127463 PMC4856451

[b22] Wang J. . The structural alteration of gut microbiota in low-birth-weight mice undergoing accelerated postnatal growth. Sci. Rep. 6, 27780 (2016). 27277748 10.1038/srep27780 PMC4899793

[b23] Krych L., Hansen C. H. F., Hansen A. K., van den Berg F. W. J. & Nielsen D. S. Quantitatively different, yet qualitatively alike: a meta-analysis of the mouse core gut microbiome with a view towards the human gut microbiome. PLoS One 8, e62578 (2013). 23658749 10.1371/journal.pone.0062578 PMC3641060

[b24] Zeng B. . Effects of age and strain on the microbiota colonization in an infant human flora-associated mouse model. Curr. Microbiol. 67, 313–21 (2013). 23604540 10.1007/s00284-013-0360-3

[b25] Friswell M. K. . Site and strain-specific variation in gut microbiota profiles and metabolism in experimental mice. PLoS One 5, e8584 (2010). 20052418 10.1371/journal.pone.0008584 PMC2798964

[b26] Xiao L. . A catalog of the mouse gut metagenome. Nat. Biotechnol. 33, 1103–8 (2015). 26414350 10.1038/nbt.3353

[b27] Sofi M. H. . pH of drinking water influences the composition of gut microbiome and type 1 diabetes incidence. Diabetes 63, 632–44 (2014). 24194504 10.2337/db13-0981 PMC3900548

[b28] Wolf K. J. . Consumption of acidic water alters the gut microbiome and decreases the risk of diabetes in NOD mice. J. Histochem. Cytochem. 62, 237–50 (2014). 24453191 10.1369/0022155413519650 PMC3966285

[b29] Sasada T. . Chlorinated Water Modulates the Development of Colorectal Tumors with Chromosomal Instability and Gut Microbiota in Apc-Deficient Mice. PLoS One 10, e0132435 (2015). 26186212 10.1371/journal.pone.0132435 PMC4505894

[b30] Bangsgaard Bendtsen K. M. . Gut microbiota composition is correlated to grid floor induced stress and behavior in the BALB/c mouse. PLoS One 7, e46231 (2012). 23056268 10.1371/journal.pone.0046231 PMC3462757

[b31] Desbonnet L. . Gut Microbiota Depletion from Early Adolescence in Mice: Implications for Brain and Behaviour. Brain. Behav. Immun., doi: 10.1016/j.bbi.2015.04.004 (2015). 25866195

[b32] David J. M., Knowles S., Lamkin D. M. & Stout D. B. Individually ventilated cages impose cold stress on laboratory mice: a source of systemic experimental variability. J. Am. Assoc. Lab. Anim. Sci. 52, 738–44 (2013). 24351762 PMC3838608

[b33] McCafferty J. . Stochastic changes over time and not founder effects drive cage effects in microbial community assembly in a mouse model. ISME J. 7, 2116–25 (2013). 23823492 10.1038/ismej.2013.106 PMC3806260

[b34] Hansen A. K., Friis Hansen C. H., Krych L. & Nielsen D. S. Impact of the gut microbiota on rodent models of human disease. World J. Gastroenterol. 20, 17727–17736 (2014). 25548471 10.3748/wjg.v20.i47.17727 PMC4273123

[b35] Hansen A. K., Krych Ł., Nielsen D. S. & Hansen C. H. F. A Review of Applied Aspects of Dealing with Gut Microbiota Impact on Rodent Models. ILAR J. 56, 250–64 (2015). 26323634 10.1093/ilar/ilv010

[b36] Macpherson A. J. & McCoy K. D. Standardised animal models of host microbial mutualism. Mucosal Immunol. 8, 476–486 (2015). 25492472 10.1038/mi.2014.113 PMC4424382

[b37] Ivanov I. I. . Induction of intestinal Th17 cells by segmented filamentous bacteria. Cell 139, 485–98 (2009). 19836068 10.1016/j.cell.2009.09.033 PMC2796826

[b38] Ericsson A. C. . Effects of vendor and genetic background on the composition of the fecal microbiota of inbred mice. PLoS One 10, e0116704 (2015). 25675094 10.1371/journal.pone.0116704 PMC4326421

[b39] Jakobsson H. E. . The composition of the gut microbiota shapes the colon mucus barrier. EMBO Rep. 16, 164–77 (2015). 25525071 10.15252/embr.201439263 PMC4328744

[b40] Ericsson A. C. . Differential susceptibility to colorectal cancer due to naturally occurring gut microbiota. Oncotarget 6, 33689–33704 (2015). 26378041 10.18632/oncotarget.5604 PMC4741795

[b41] Celaj S. . The microbiota regulates susceptibility to Fas-mediated acute hepatic injury. Lab. Invest. 94, 938–49 (2014). 25068658 10.1038/labinvest.2014.93 PMC4152405

[b42] Chang H.-Y. S., Mitzner W. & Watson J. Variation in airway responsiveness of male C57BL/6 mice from 5 vendors. J. Am. Assoc. Lab. Anim. Sci. 51, 401–6 (2012). 23043804 PMC3400687

[b43] Hoy Y. E. . Variation in Taxonomic Composition of the Fecal Microbiota in an Inbred Mouse Strain across Individuals and Time. PLoS One 10, e0142825 (2015). 26565698 10.1371/journal.pone.0142825 PMC4643986

[b44] Lundberg R. . Gastrointestinal microbiota and local inflammation during oxazolone-induced dermatitis in BALB/cA mice. Comp. Med. 62, 371–80 (2012). 23114040 PMC3472601

[b45] Ellekilde M. . Characterization of the gut microbiota in leptin deficient obese mice - Correlation to inflammatory and diabetic parameters. Res. Vet. Sci. 96, 241–50 (2014). 24556473 10.1016/j.rvsc.2014.01.007

[b46] Theriault B. . Long-term Maintenance of Sterility Following Skin Transplantation in Germ-free Mice. Transplant. direct 1, (2015). 10.1097/TXD.0000000000000539 PMC4655119 26609546

