# Supplementary material for: Microbiota composition of simultaneously colonized mice housed under either a gnotobiotic isolator or individually ventilated cage regime
Source: Sci Rep. 2017 Feb 7;7:42245. doi: 10.1038/srep42245 (PMC5294411; doi:10.1038/srep42245)
Supplement: Supplementary Information [file srep42245-s1.pdf]

Supplementary Information

Microbiota composition of simultaneously colonized mice housed under either a gnotobiotic isolator or individually ventilated cage regime

Randi Lundberg[1,2*], Martin I. Bahl[3], Tine R. Licht[3], Martin F. Toft[2,4], Axel K. Hansen[1]

[1]Section of Experimental Animal Models, Department of Veterinary Disease Biology, Faculty of Health and Medical Sciences, University of Copenhagen, 1871 Frederiksberg C, Denmark

[2]Internal Research and Development, Taconic Biosciences, 4623 Lille Skensved, Denmark

[3]National Food Institute, Technical University of Denmark, 2860 Søborg, Denmark

[4]Current address: Adlast, 6200 Aabenraa, Denmark

*Corresponding author: randi.lundberg@taconic.com

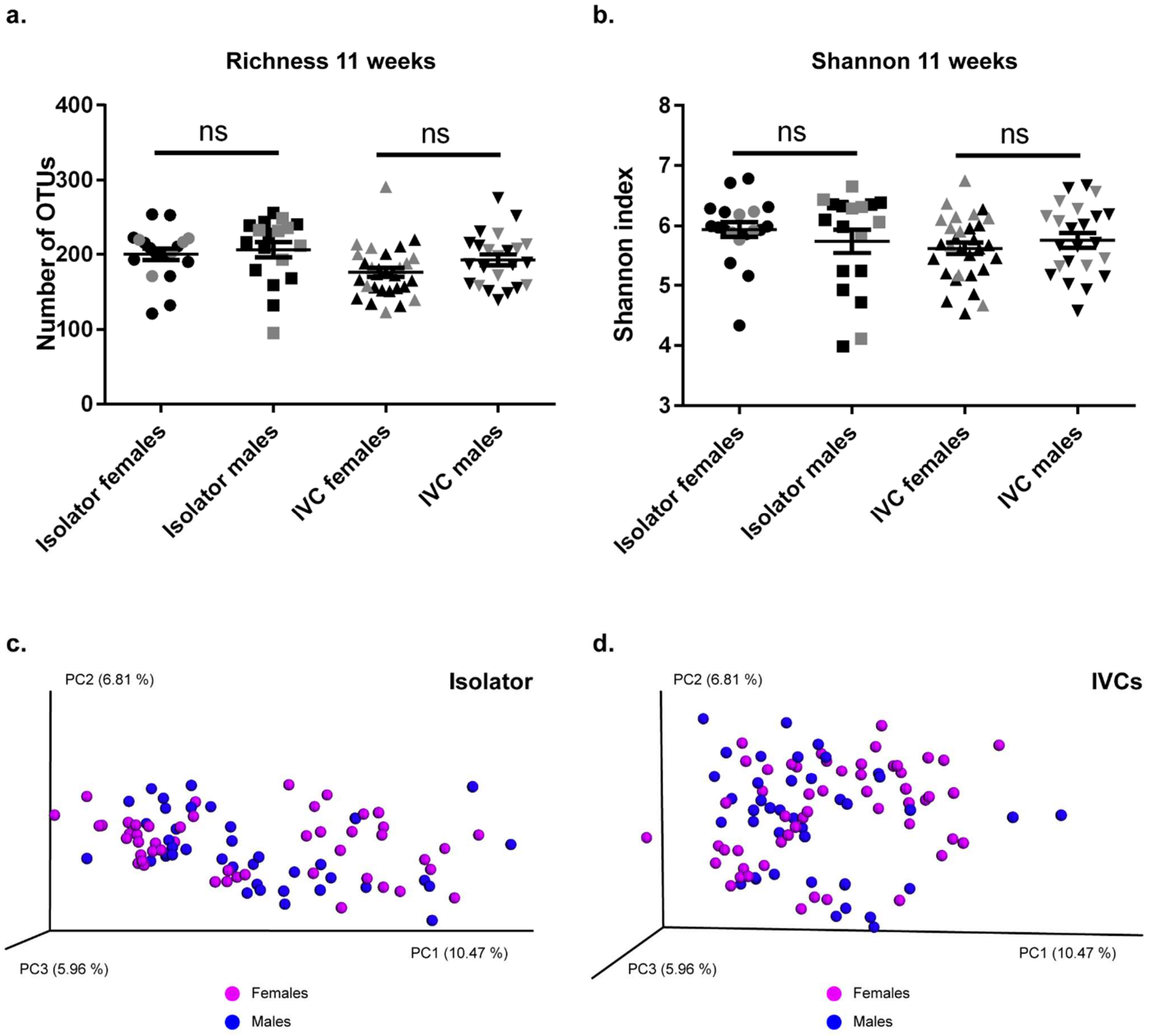

Supplementary Figure S1. Alpha and beta diversity of the gut microbiota of isolator- and IVC-housed mice according to sex. a+b. Richness **(a)** and Shannon index **(b)** of fecal samples from female and male mice 11 weeks old in the isolator and IVCs. Black symbols=F1 mice; Grey symbols=F2 mice. There were no significant differences between the microbiotas from male or female mice (t-test, s.e.m.). **c+d.** Unweighted 3D UniFrac Principal Coordinate Analysis of fecal samples from isolator **(c)** and IVC **(d)** mice 11 weeks old. There was no significant clustering between the microbiotas from male and female mice (Isolator: p=0.269, R=0.01; IVCs: p=0.524, R=-0.00; ANOSIM with 999 permutations).

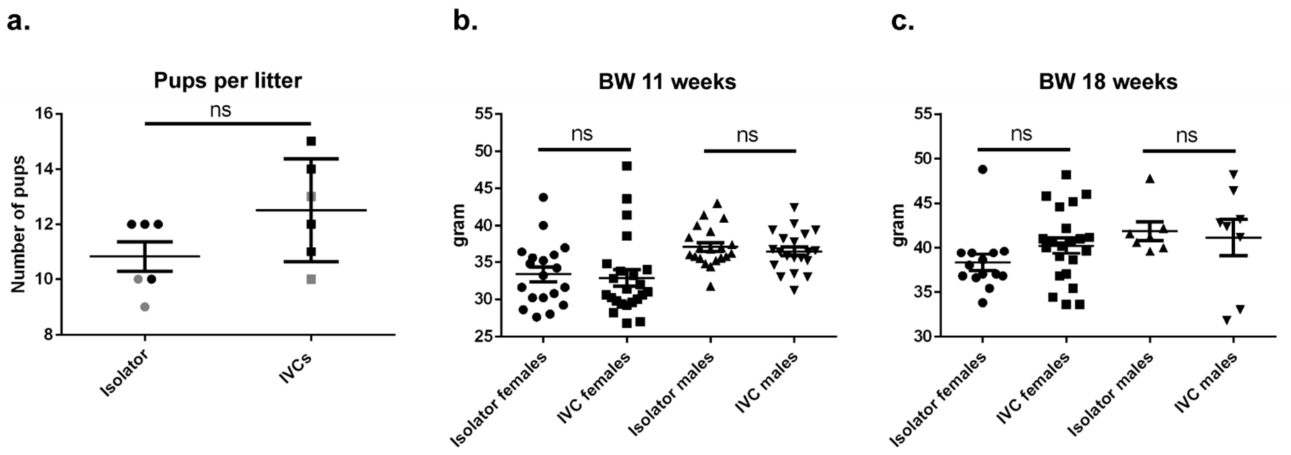

Supplementary Figure S2. Pups per litter and body weight of isolator- and IVC-housed mice. a. Pups per litter. Black symbols=F1 litters; Grey symbols=F2 litters. There was no significant difference between the isolator and IVC-housed mice. **b+c.** Body weight at termination for 11 **(b)** and 18 **(c)** weeks old mice. There were no significant differences between body weight in the two housing systems (t-test, s.e.m.).

Hypothesis	p-value	95% CI of the difference
**Isolator**		
**P-7 wk vs. F1-18 wk**	0.038	(-0.3964; -0.0062)
**F1-4 wk vs. F1-18 wk**	0.018	(-0.2233; -0.0126)
**F2-4 wk vs. F1-11 wk**	0.001	(-0.2883; -0.0461)
**F2-4 wk vs. F1-18 wk**	<0.0001	(-0.3232; -0.0663)
**F2-4 wk vs. F2-11 wk**	0.010	(-0.3000; -0.0252)
**IVCs**		
**P-7 wk vs. F1-18 wk**	0.061(*)	(-0.2859; 0.0033)
**F1-4 wk vs. F1-11 wk**	<0.0001	(-0.1409; -0.0269)
**F1-4 wk vs. F1-18 wk**	<0.0001	(-0.1643; -0.0357)
**F2-11 wk vs. F1-4 wk**	0.002	(0.0210; 0.1556)
**F2-4 wk vs. F1-11 wk**	<0.0001	(-0.1901; -0.0445)
**F2-4 wk vs. F1-18 wk**	<0.0001	(-0.2121; -0.0547)
**F2-4 wk vs. F2-11 wk**	<0.0001	(-0.2028; -0.0405)

Supplementary Table S1. Colonization efficiency comparisons between different generations and ages of isolator- and IVC-housed mice (related to Figure 2a). The colonization efficiency, i.e. the number of shared taxa on genus level of each generation and age compared to inoculum, was calculated and differences between all pairs of groups (P 7, 11 and 18 weeks, F1 4, 11 and 18 weeks, and F2 4 and 11 weeks) were tested (ANOVA with Tukey's pairwise comparisons). Only tests with a p-value <0.08 is shown here. We considered a p-value >0.05 and <0.08 as a tendency and is designated with (*). Wk=weeks. CI=confidence interval.

Hypothesis	Shannon p-value	Shannon 95% CI of the difference	Richness p-value	Richness 95% CI of the difference
**IVCs**				
**4 vs. 7 wk**	0.987	(-1.105; 0.855)	1.000	(-58.5; 54.3)
**4 vs. 11 wk**	0.264	(-0.549; 0.096)	0.001*	(-45.56; -8.46)
**4 vs. 18 wk**	0.073(*)	(-0.797; 0.024)	0.000*	(-66.91; -19.65)
**7 vs. 11 wk**	0.784	(-1.329; 0.625)	0.533	(-85.4; 27.1)
**7 vs. 18 wk**	0.552	(-1.521; 0.497)	0.181	(-103.5; 12.7)
**11 vs. 18 wk**	0.730	(-0.242; 0.562)	0.264	(-6.87; 39.41)
**Isolator**				
**4 vs. 7 wk**	0.262	(-0.317; 1.806)	0.397	(-34.2; 82.6)
**4 vs. 11 wk**	0.051(*)	(-0.876; 0.002)	0.000*	(-65.72; -17.39)
**4 vs. 18 wk**	0.108	(-0.964; 0.065)	0.003*	(-66.9; -10.2)
**7 vs. 11 wk**	0.867	(-0.738; 1.353)	0.858	(-74.9; 40.2)
**7 vs. 18 wk**	0.890	(-0.785; 1.374)	0.921	(-73.8; 45.1)
**11 vs. 18 wk**	1.000	(-0.469; 0.494)	0.991	(-29.5; 23.5)

Supplementary Table S2. Alpha diversity of different ages of isolator- and IVC-housed mice (related to Figure 3). Shannon index and richness were analysed between different ages in each housing system (ANOVA with Tukey's pairwise comparisons). We considered a p-value >0.05 and <0.08 as a tendency and is designated with (*). P-values <0.05 are designated with *. Wk=weeks. CI=confidence interval.

Phylum	Genus	Inoc.	Isolator								IVCs								
			P			F1			F2		P			F1			F2		
			7 wk	11 wk	18 wk	4 wk	11 wk	18 wk	4 wk	11 wk	7 wk	11 wk	18 wk	4 wk	11 wk	18 wk	4 wk	11 wk	
Bacteroidetes	Uncl. S24-7	20.8	3.2	51.9	32.7	31.0	20.4	17.3	22.0	16.6	19.7	45.0	14.8	19.1	21.0	13.8	9.2	19.0	%
	Alistipes	5.8	22.9	12.3	11.7	12.2	10.4	15.4	6.7	16.1	26.6	18.8	5.2	13.0	11.0	11.6	5.0	10.3	%
	Bacteroides	0.4	12.1	3.8	1.1	5.7	4.7	1.8	5.0	2.2	5.0	1.0	0.7	4.0	1.9	1.9	1.6	3.9	%
	Odoribacter	0.4	2.1	2.0	1.0	2.3	0.9	1.2	1.1	1.1	4.7	1.2	0.8	2.0	1.3	1.0	1.4	1.7	%
	RC9 gut group	0.1	0.0	0.0	0.0	0.0	0.0	0.0	0.0	0.4	3.9	1.4	1.4	6.9	2.5	1.5	0.9	4.1	%
	Uncl. Prevotellaceae	1.1	3.5	0.9	0.5	2.1	0.5	0.5	1.5	0.4	4.7	2.1	0.2	2.1	1.4	0.9	2.0	2.0	%
Firmicutes	Lactobacillus	0.6	28.4	5.7	6.9	19.1	13.9	13.5	46.6	22.0	7.4	3.6	24.3	29.7	23.3	19.2	59.6	37.3	%
	Uncl. Lachnospiraceae	50.7	15.0	14.9	34.3	15.4	35.1	35.7	6.9	29.5	11.3	14.2	21.0	11.5	28.3	39.5	12.4	10.9	%
	Uncl. Ruminococcaceae	4.3	2.7	1.8	2.4	2.7	2.8	3.0	1.5	2.0	5.7	1.7	2.0	1.5	1.8	2.0	1.8	1.6	%
	SFB	0.1	2.6	0.8	0.0	2.2	0.3	0.2	2.8	0.7	1.8	4.1	0.0	4.8	0.3	0.1	0.6	3.5	%
Abundance <1%		15.7	7.5	5.8	9.4	7.3	10.9	11.3	5.9	8.9	9.1	6.9	29.8	5.3	7.3	8.7	5.3	5.7	%
**Total**		100	100	100	100	100	100	100	100	100	100	100	100	100	100	100	100	100	%

Supplementary Table S3. Gut microbiota composition of inoculum and fecal samples from isolator- and IVC-housed mice (related to Figure 4a). Relative abundance (%) of the 10 most abundant taxa and taxa with <1% abundance collapsed in the inoculum, isolator and IVC samples from P, F1 and F2 generations at various ages in weeks (wk). Uncl.=Unclassified. Inoc.=Inoculum.
